# Comparative genomic analysis of Planctomycetota potential for polysaccharide degradation identifies biotechnologically relevant microbes

**DOI:** 10.1186/s12864-024-10413-z

**Published:** 2024-05-27

**Authors:** Dominika Klimek, Malte Herold, Magdalena Calusinska

**Affiliations:** 1 Environmental Research and Innovation Department, Luxembourg Institute of Science and Technology (LIST), 41 rue du Brill, Belvaux, L-4422 Luxembourg; 2 The Faculty of Science, Technology and Medicine (FSTM), University of Luxembourg, 2 Avenue de l’Université, Esch-sur-Alzette, L-4365 Luxembourg

**Keywords:** Planctomycetota, Carbohydrate-active enzymes (CAZymes), Carbohydrolytic potential, Algal and lignocellulosic biomass degradation, Bioprospecting

## Abstract

**Background:**

Members of the *Planctomycetota* phylum harbour an outstanding potential for carbohydrate degradation given the abundance and diversity of carbohydrate-active enzymes (CAZymes) encoded in their genomes. However, mainly members of the *Planctomycetia* class have been characterised up to now, and little is known about the degrading capacities of the other *Planctomycetota*. Here, we present a comprehensive comparative analysis of all available planctomycetotal genome representatives and detail encoded carbohydrolytic potential across phylogenetic groups and different habitats.

**Results:**

Our in-depth characterisation of the available planctomycetotal genomic resources increases our knowledge of the carbohydrolytic capacities of *Planctomycetota*. We show that this single phylum encompasses a wide variety of the currently known CAZyme diversity assigned to glycoside hydrolase families and that many members encode a versatile enzymatic machinery towards complex carbohydrate degradation, including lignocellulose. We highlight members of the *Isosphaerales, Pirellulales, Sedimentisphaerales* and *Tepidisphaerales* orders as having the highest encoded hydrolytic potential of the *Planctomycetota*. Furthermore, members of a yet uncultivated group affiliated to the *Phycisphaerales* order could represent an interesting source of novel lytic polysaccharide monooxygenases to boost lignocellulose degradation. Surprisingly, many *Planctomycetota* from anaerobic digestion reactors encode CAZymes targeting algal polysaccharides – this opens new perspectives for algal biomass valorisation in biogas processes.

**Conclusions:**

Our study provides a new perspective on planctomycetotal carbohydrolytic potential, highlighting distinct phylogenetic groups which could provide a wealth of diverse, potentially novel CAZymes of industrial interest.

**Supplementary Information:**

The online version contains supplementary material available at 10.1186/s12864-024-10413-z.

## Introduction

Modern society generates enormous amount of waste organic matter that requires specific and well-defined disposal procedures [1]. Instead, waste biomass could be valorised into added-value products and energy [2]. In the light of global threats like environmental pollution and climate change, the bioconversion of organic waste into biofuels and sustainable, added-value products has been gaining considerable attention [3]. Microorganisms possess a broad repertoire of hydrolytic enzymes for aerobic and anaerobic degradation of organic matter [4]. The enzymes involved in carbohydrate breakdown are known as carbohydrate-active enzymes (CAZymes) and are currently classified into five classes that include glycoside hydrolases (GH), carbohydrate esterases (CE), polysaccharide lyases (PL) and enzymes with auxiliary activities (AA) [5]. Carbohydrate binding modules (CBMs) are non-catalytic modules, generally defined as accessory CAZymes, and their main role is to recognise the substrates by binding carbohydrates [6]. Several different industrial sectors, such as food industries and biorefineries, rely on the application of CAZymes from bacterial and fungal strains [7]. The increasing availability of genomic data provides promising avenue to discover novel strains and enzymes for scientific and industrial applications for e.g., heterologous expression [8]. Although a number of metagenomic studies have revealed a high diversity of microorganisms capable of degrading complex polysaccharides in distinct biomass-rich habitats, little attention has been paid to *Planctomycetota* [4, 9, 10]. According to a recent analysis of the global distribution of carbohydrate utilisation potential in the tree of life, alongside *Bacteroidota* and a few other phyla, *Planctomycetota* was identified as one of the most versatile phyla in degrading diverse biopolymers of cellulosic and non-cellulosic origin [11]. *Planctomycetota*, previously known as *Planctomycetes*, is one of the phyla within the *Planctomycetota-Verrucomicrobiota-Chlamydiota* superphylum (PVC). They are characterised with distinctive features not commonly detected in other prokaryotes, such as enlarged periplasm, outer membrane complexes in the form of crateriform structures, and a non-FtsZ based division mode [12, 13]. Besides these cellular particularities, bacteria belonging to this widespread phylum have been highlighted in different environments for their hydrolytic potential [14]. Uncultured *Planctomycetota* have been identified as primary degraders of extracellular polymeric substances in soil and complex carbohydrates in marine sediments [15–17]. Certain members of this phylum can attach to algal surfaces and have been proposed to depolymerise algae-derived polymers [18, 19]. Accordingly, *Rhodopirellula* is widely known for producing multiple and diverse sulphatases engaged in sulphated polysaccharides degradation like algal polysaccharides [20]. Metatranscriptomic studies also revealed the contribution of *Planctomycetota* to complex polysaccharide degradation in *Sphagnum*-dominated areas [21]. However, so far, only a limited number of *Planctomycetota* have been identified as potential candidates for biotechnological applications such as the bioactive compound production [22], and most of the characterised strains derive from the *Planctomycetia* class only [23, 24].

In this study we investigate the encoded carbohydrolytic capacities of *Planctomycetota*, including the *Planctomycetia* and *Phycisphaerae* classes, as well as other less characterised members of this phylum. Through bioinformatics analysis of 1425 non-redundant genomes, we unveil a number of diverse CAZymes across planctomycetotal orders, emphasising their versatile encoded capabilities in carbohydrate degradation. High incidence of CAZyme gene clusters and presence of potentially extracellular enzymes, point to the existence of coordinated strategies for complex polysaccharide degradation, including lignocellulose and algal biomasses.

## Methods

### Dataset acquisition and classification of planctomycetotal genomes

Over 3000 publicly available draft and complete planctomycetotal genomes were downloaded from GenBank in June 2021 (http://www.ncbi.nlm.nih.gov/genbank/), using ncbi-genome-download (https://github.com/kblin/ncbi-genome-download). The initial genome database was complemented with the metagenome-assembled genomes (MAGs) from own studies (see Additional File 1, Table S1 for further details) as well as genomes from the catalogue of Earth’s Microbiomes (GEM) available from the JGI [25]. Unless stated otherwise, we will refer to both individual genomes and MAGs as genomes. All genomes were assessed for redundancy using dRep v3.2.2 with the -con 10 --checkM_method taxonomy_wf parameters [26]. The resulting 1457 non-redundant genomes were classified taxonomically with GTDB-tk v1.2.0 against GTDB database release 89 [27] and only hits designated as *Planctomycetota* were retained. A few changes were introduced to the taxonomic designations. The *Planctomycetes* class was renamed *Planctomycetia* as originally proposed by NL Ward 2011 (Bergey’s Manual) and formally adopted in Oren and Garrity 2020 [28, 29]. Furthermore, the candidate order UBA1161 was renamed *Tepidisphaerales* [30], as proposed by Dedysh et al. [31]. CheckM v1.2.0 was used to determine genome completeness and contamination [32] and only genomes meeting the MIMAG standard of medium to high quality level (i.e. completeness above 75% and contamination below 10%) were retained for further analyses [33]. At this stage, our database contained 1451 non-redundant planctomycetotal genomes. We further reduced this number to 1425, by excluding the genomes encoding less than 10 carbohydrate active enzymes (CAZymes). To simplify the analysis, we designated classes with fewer than 10 sequenced genomes as “other class”. The final database of 1425 non-redundant genomes was additionally complemented with the respective metadata retrieved from NCBI using the rentrez script [34], followed by manual curation for conflicting information. Environmental metadata from the GEM catalogue was retrieved directly from the deposited repository and unified with the NCBI entries for the different habitat categories (Additional File 1, Table S2).

### Functional annotation of planctomycetotal genomes

Genomes were gene-called by prodigal v2.6.3 [35] and CAZymes were annotated by dbCAN2 v2.0.11 [36] against the dbCAN database v9 [5] using the three integrated tools (DIAMOND, Hotpep and, HMMER) with default parameters [37–39]. Genome annotation was also performed using Prokka v1.14.6 with its default databases [40]. To determine clusters of co-localised CAZymes, we applied a modified version of the CGCFinder module of dbCAN2 to detect CAZyme gene clusters (CGCs) [36]. CGCs were predicted as consisting of at least one CAZyme coding gene with at least one auxiliary gene (e.g. transcription factor or transporter) or another CAZyme separated by at most two other genes. Positive hits were assigned to CAZyme families if annotated by HMMER v3.1.2 and multiple CAZyme assignments were considered as separate functional domains or modules. For searching putatively novel CAZymes, only hits annotated either by DIAMOND v0.9.19 or Hotpep, but not HMMER, were retained (as so-called unclassified CAZymes). To assess the novelty of predicted CAZymes (assigned by HMMER), we searched the protein sequences against the CAZy database with DIAMOND and amino acid sequence identity of the best hit was inferred. Signal peptides were detected using signalP v6 [41]. Glycosyltransferase coding genes were excluded from the analysis as they are not involved in polysaccharide degradation. The raw output files of dbCAN2, CGCFinder and signalP are available in Additional File 4, 5, and 6, respectively.

### Data analysis

Statistical analyses and visualisations were performed with the R software v 4.0.2 [42]. For multivariate analyses, a presence-absence table of CAZyme content for each genome was transformed into a Jaccard distance matrix (Additional File 2, Table S3). CAZyme dissimilarity was assessed using principal coordinate analysis (PCoA) and permutational ANOVA (PERMANOVA) as well as analysis of similarities (ANOSIM) with the vegan v 2.5.7 package in R [43]. Linear discriminant analysis (LDA) was performed with a nonparametric Kruskal Wallis test using the microbial v0.0.22 package in R (logarithmic LDA score > 4) [44]. A phylogenetic tree of genomes was constructed from the alignment of default marker genes using PhyloPhlAn v3.0.60 (--diversity medium supertree_aa) [45]. The alignment of protein sequences was calculated using the MUSCLE algorithm with default parameters [46]. Pairwise comparisons between protein sequences and Neighbor-Joining consensus were calculated for constructing the tree using Geneious Prime v 2019.0.3 [47]. The Spearman’s rank correlation was calculated in R using package stats. Unless otherwise stated, the significance of differences between tested groups was assessed using either a non-parametric Kruskal-Wallis or Wilcoxon test (R package stats). The obtained *p*-values were adjusted for multiple testing using the Benjamini–Hochberg procedure (false-discovery rate).

### Annotation of CAZyme family activities

The substrate database (CAZyme families assigned to substrates) was framed according to [11] and the CAZy database [5] (Additional File 3). For CAZyme functional analysis, entries assigned to GH and PL families were classified based on their main characterised enzymatic activities into four categories according to the main target: algal biomass (algae-derived polymers), plant biomass (plant storage polysaccharides, oligosaccharides, and cellulose-hemicellulose fractions), algal/plant biomass and other activities (all the remaining polysaccharide targets were grouped together). Further, the categories were subdivided based on the substrate specificity: algal polysaccharides, glucans (α- and β-glucans), oligosaccharides, lignocellulose (cellulosic and/or hemicellulosic backbone), NAG-based polysaccharides (based on N-acetylglucosamine, including bacterial and host glycans), pectin, and other polysaccharides. The detailed annotations of substrates are available in the Additional File 3. The ratio of CAZymes for polysaccharide target specificity was calculated by comparing the number of CAZymes (GHs and/or PLs) with assigned function to the number of all predicted CAZymes (GHs and/or PLs).

## Results and discussion

### Database of planctomycetotal genomes

**Fig. 1:**
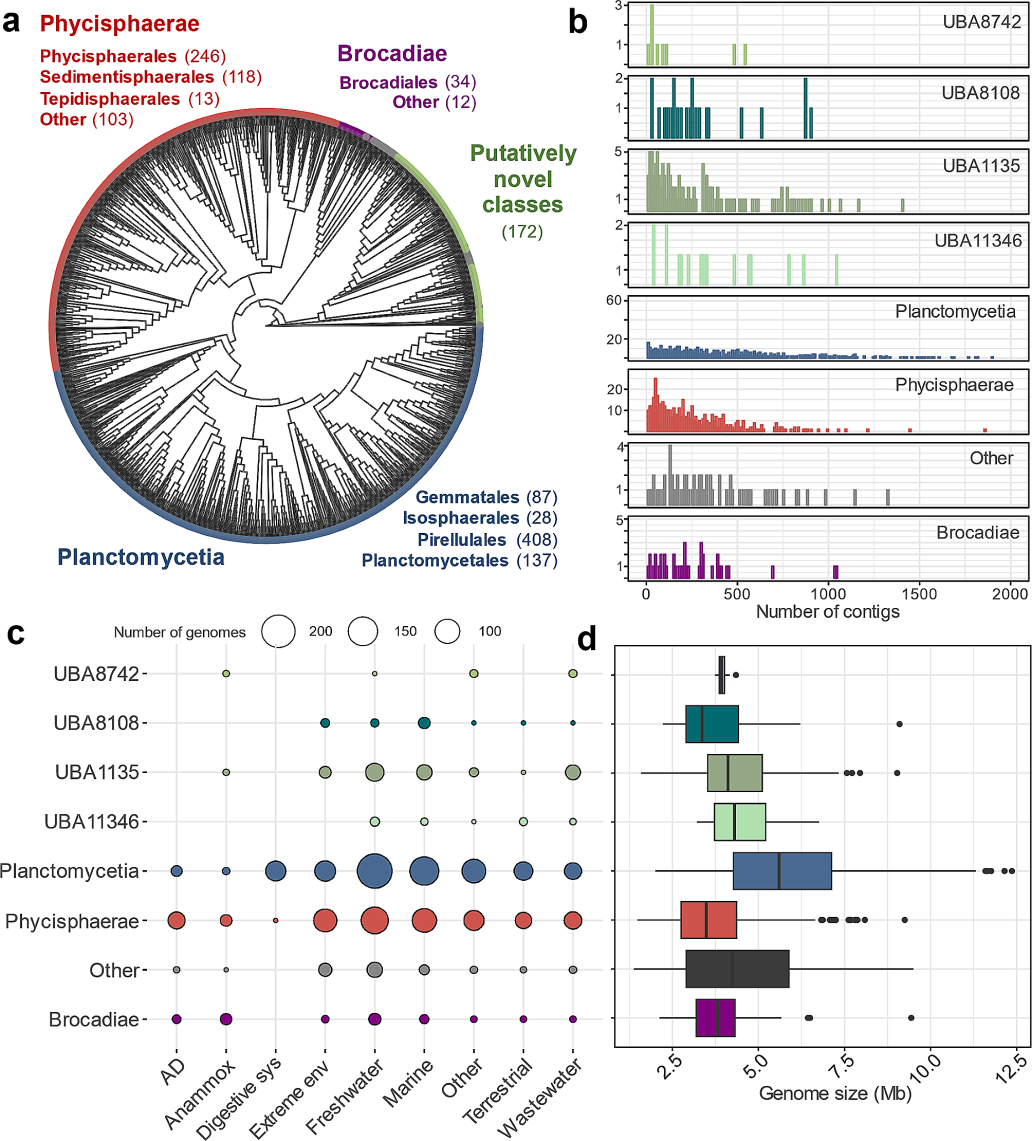
Overview of planctomycetotal genomes included in the study, grouped and coloured at the class level. (**a**) The phylogenetic distribution of planctomycetotal genomes. The grey colour on the outer circle represents genomes assigned to “other class”. (**b**) Histogram of the genome fragmentation level **c-d.** Environmental origin, further called “habitat” (**c**) and genome size in Mb (**d**) of planctomycetotal genomes

In this study, we investigated the metabolic potential of *Planctomycetota* for polysaccharide degradation. We tried to identify the primary trends across *Planctomycetota* by concentrating the analysis on the class and order taxonomic levels (Fig. 1). We argue that lower than phylum taxonomic level genomic comparisons provide a more nuanced and detailed perspective on the carbohydrolytic potential, enabling us to investigate common patterns that may not be as evident when carrying out a comparison at the bacterial phylum levels only.

To characterise the carbohydrate degrading potential of *Planctomycetota*, we created a database of 1425 non-redundant and medium to high quality genomes of different fragmentation level, recovered from both metagenomics and isolate sequencing studies (Fig. 1a-d; Additional File 1, Table S1). Our database reflects all currently known as well as putatively novel classes of *Planctomycetota* (Fig. 1a), allowing us to largely complement another recent study of microbial CAZymes, which included only 243 planctomycetotal genomes [11]. Specifically, the database includes 662 genomes of the *Planctomycetia* class with the following orders: *Gemmatales* (number of genomes, *n* = 87), *Isosphaerales* (*n* = 28), *Pirellulales* (*n* = 408), and *Planctomycetales* (*n* = 137). Furthermore, it includes 463 genomes of the *Phycisphaerae* class including the *Phycisphaerales* (*n* = 246), *Sedimentisphaerales* (*n* = 118), and *Tepidisphaerales* (*n* = 13), as well as putative UBA1845 (*n* = 64) and SM23-33 (*n* = 22) orders. *Planctomycetia* and *Phycisphaerae* are the two biggest and widely described classes of the *Planctomycetota* phylum, and a few isolated representatives are the only so far cultured and characterised carbohydrate degrading *Planctomycetota* [13]. Additionally, 46 genomes of the *Brocadiae* candidate class are included, which are commonly known as anaerobic ammonium oxidising (anammox) bacteria widely employed in wastewater treatment settings [48]. Other genomes (*n* = 172) represent novel, not yet assigned planctomycetotal classes, including UBA8742, UBA8108, UBA1135 and UBA11346, which we labelled “putatively novel classes” (Fig. 1a). Genomes that represent other less populated classes of *Planctomycetota* (< 10 genomes) were grouped together as “other class” (see Methods, *n* = 65).

According to the environmental metadata, half of the planctomycetotal genomes in our database originate from marine and freshwater habitats (51%) while the remaining genomes were retrieved from extreme environments including thermal springs, hydrothermal vents and saline/alkaline habitats (13%), wastewater (8%), terrestrial (7%), animal digestive systems (4%), anammox (2%), AD reactors (4%) and other environments (11%) (Fig. 1c; Additional File 1, Table S2).

### Phylum- and class-level distribution of planctomycetotal CAZymes

**Fig. 2:**
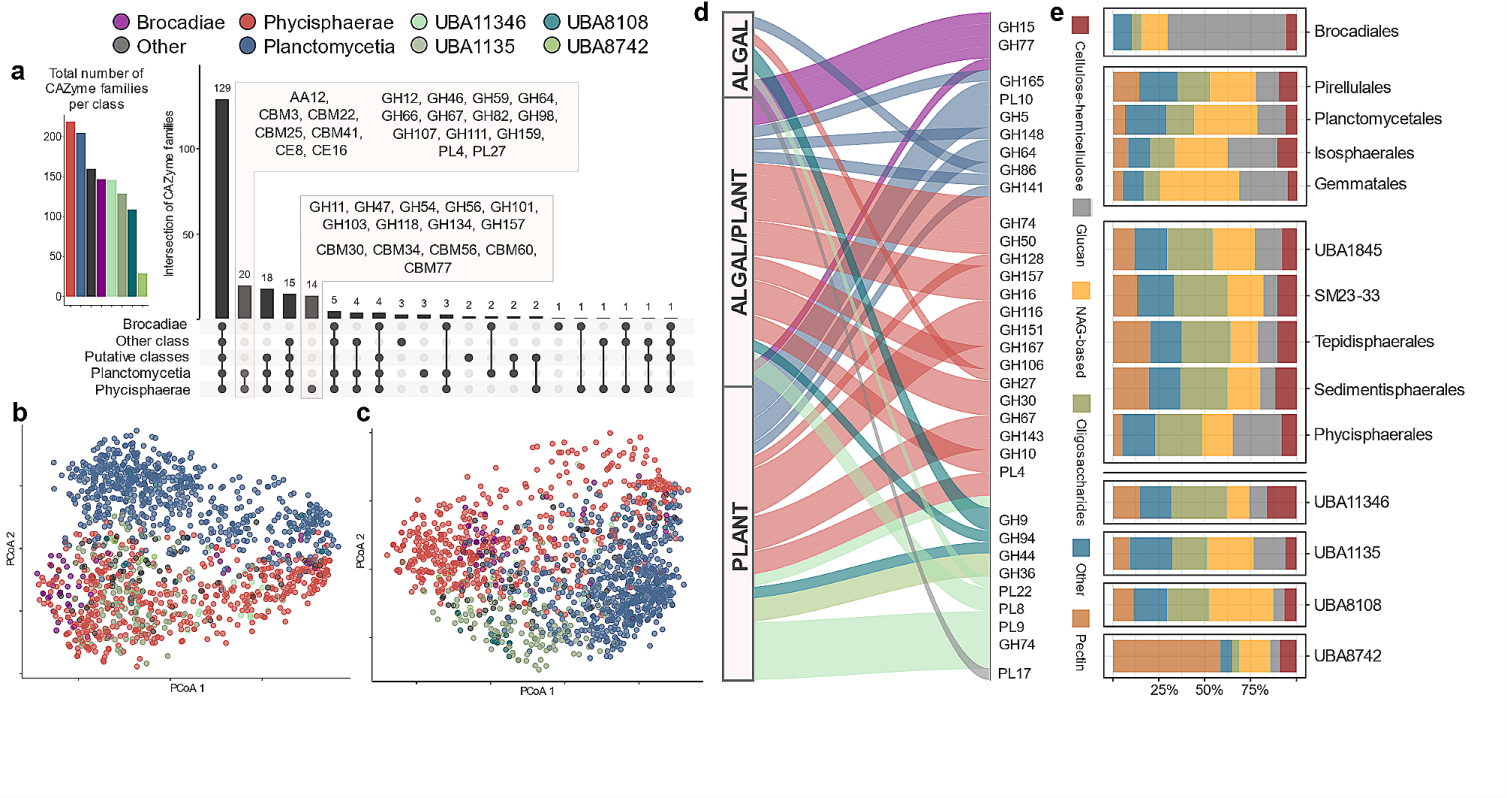
Characterisation of planctomycetotal CAZyomes (CAZyme repertoires) coloured by class affiliation. For **a, b, c** and **d**, the colour code corresponds to planctomycetotal classes, as indicated at the top of the figure. **a.** Bar plot (left) and upset plot (right), representing the number of CAZyme families and intersections between the planctomycetotal classes. **b-c.** Principal coordinates analysis (PCoA) ordination based on the Jaccard distance presence-absence matrix of GHs (**b**, ANOSIM = 0.45) and all the other CAZyme families (**c**, ANOSIM = 0.41) encoded in planctomycetotal genomes. **d.** Alluvial plot representing the number of significantly enriched GH and PL families in planctomycetotal orders (*p* < 0.05) with assigned functions towards either type of biomass. Only selected CAZyme families are highlighted. **e.** Bar plot representing the functional assignment of GHs and PLs, grouped at the order level and coloured by the substrate category

The complete deconstruction of polysaccharides requires GH interaction with other CAZymes, including PLs responsible for the non-hydrolytic cleavage of glycosidic bonds and carbohydrate esters hydrolysing CEs, as well as other redox enzymes with auxiliary activities, such as AAs and including the lytic polysaccharide monooxygenases (LPMOs) [49]. Therefore, we first assessed the set of CAZyme families in the *Planctomycetota* genomes to estimate their catalytic potential. Globally, we detected 232 CAZyme families and 132 CAZyme subfamilies (Additional File 2, Table S3), demonstrating that this phylum alone covers 80% of the known GH family diversity at the time of analysis (September 2022). In turn, the diversity of AAs, CEs, PLs and CBMs represents 53%, 70%, 69% and 43% of the family diversity described, respectively. By examining the distribution of CAZymes across planctomycetotal classes, we found 129 CAZyme families that are shared between all the classes of *Planctomycetota* (Fig. 2a). The *Phycisphaerae* class displays the greatest encoded diversity, including unique families such as β-agarases GH118, mannan-targeting GH47 and GH134, xylanases GH11 and α-L-arabinofuranosidases GH54 (Fig. 2a). Conversely, the CAZyme families in UBA8742 exhibit little diversity, but families including GH44, putatively engaged in hemicellulose degradation, and pectin-targeting PL9 are frequently encoded in representative genomes of this class (Additional File 7, Fig. S1). Genomes belonging to the *Planctomycetia* class are deprived of genes that encode GH102 (peptidoglycan lyase), which are present in all other classes of *Planctomycetota* (Additional File 7, Fig. S1). Certain planctomycetotal genomes encode CAZymes assigned to the AA12 family representing putative oxidoreductases; which have never before been detected in any prokaryote [5], thus representing an interesting avenue for future studies.

To further compare CAZyomes at the planctomycetotal class level, we employed principal coordinate analysis (PCoA, Fig. 2b-c) applied to the CAZyme occurrence matrix. We observed a moderate separation between the different planctomycetotal classes, especially visible for GH families (Fig. 2b), which was further supported by statistical tests (PERMANOVA *p* < 0.01 and ANOSIM *R* = 0.45 *p* < 0.01). We found that genome origin (habitat) has only a low impact on the carbohydrate degrading potential (Additional File 7, Fig. S2; ANOSIM *R* = 0.06 *p* < 0.01).

### Redundant hydrolytic potentials of distinct planctomycetotal orders

To specifically assess the differences in the carbohydrate degrading potentials, we further detected enriched CAZyme families in planctomycetotal genomes, identifying a panel of 101 differentially encoded CAZymes within the *Planctomycetota* orders (Fig. 2d; Additional File 2, Table S4). Considering the fact that different GH families may catalyse the hydrolysis of structurally similar substrates and seemingly diverse CAZyomes could be functionally redundant [50], we broadly classified the differentially enriched CAZymes in planctomycetotal genomes into different biomass and substrate categories (Fig. 2e). These functional categories were assigned to CAZyme families based on the main described prevailing enzyme activities (see Methods). However, this approach may be limited by the broad diversity of catalytic activities within known families, particularly GHs. Future investigations should be supplemented with more detailed bioinformatic approaches and experimental validation.

**Fig. 3:**
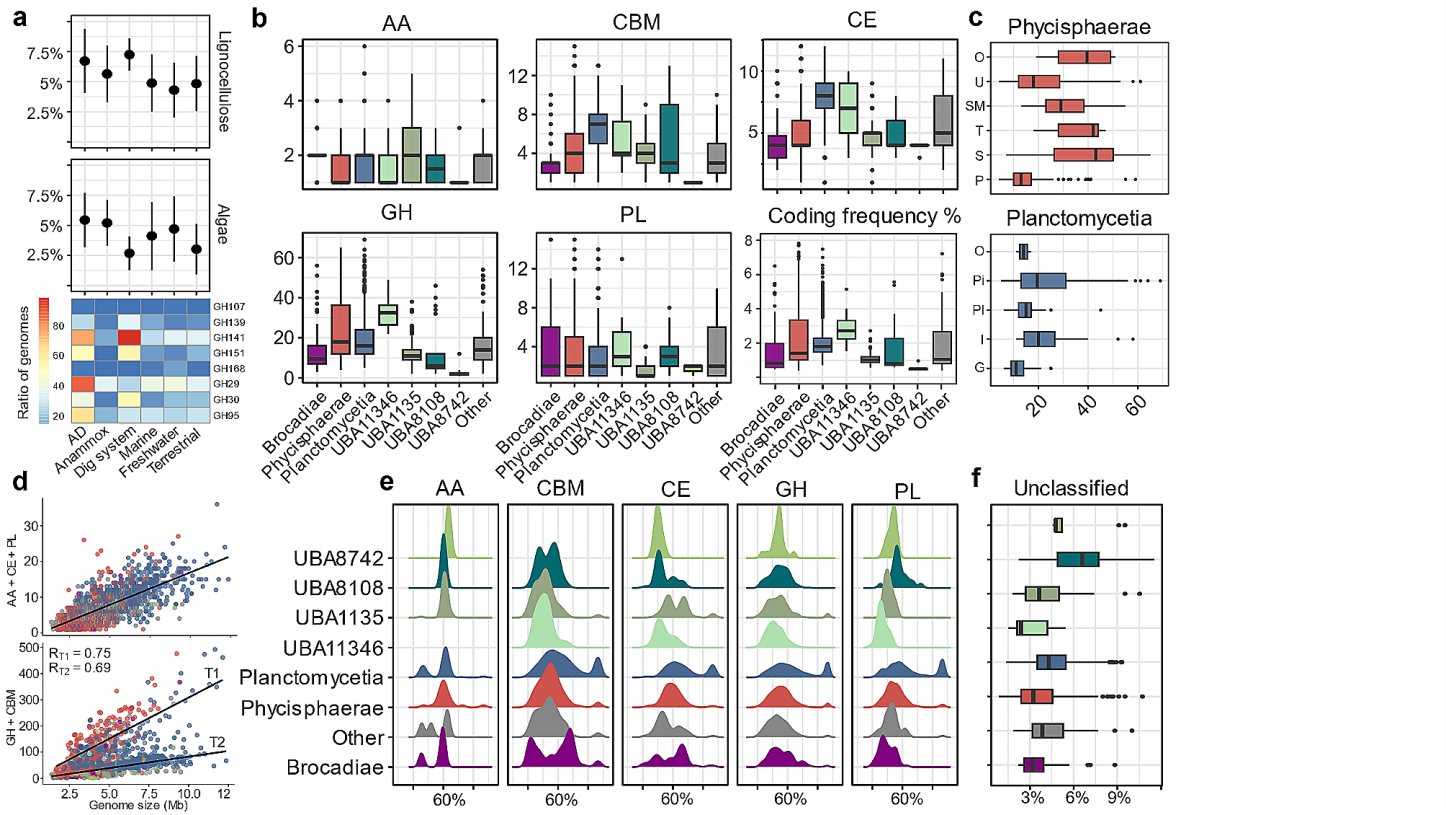
CAZyme family diversity, coding frequency and protein sequence identity of planctomycetotal genomes coloured by class affiliation. (**a**) Ratio of algalytic and lignocellulolytic CAZymes encoded by individual planctomycetotal genomes, grouped by the environmental origin. The heatmap illustrates the number of genomes encoding the listed CAZyme families. (**b**) CAZyme family diversity at the class level. Bottom right panel: CAZyme coding frequency (ratio of CAZymes to protein-coding genes) at the class level. (**c**) GH family diversity at the order level for two main classes of *Planctomycetota.* Phycisphaeral orders from the top: O – Other, U – UBA1845, SM – SM23-33, T – Tepidisphaerales, S – Sedimentisphaerales, P – Phycisphaerales; Planctomycetial orders from the top: O – Other, Pi – Pirellulales, Pl – Planctomycetales, I – Isosphaerales, G – Gemmatales. (**d**) Genome size versus number of CAZyme coding genes for each planctomycetotal genome. Trends for GH + CBM families were established based on the thresholds for low (T2, < 2%) and medium to high (T1, > 2%) CAZyme coding frequencies. (**e**) CAZyme protein sequence identity to public databases. (**f**) Ratio of unclassified CAZymes

While different CAZyme families are preferentially encoded in different groups with notable differences between taxonomic orders, all *Planctomycetota* seem equally well equipped for the degradation of main biomass and substrate categories, regardless of their phylogenetic origin and habitat specificity. Interestingly, the planctomycetotal genomes of marine bacteria are enriched with lignocellulose-degrading CAZymes (Fig. 3a), even though marine polysaccharides differ from terrestrial carbohydrates, and are often highly sulphated, especially in algal polysaccharides [51]. Planctomycetotal genomes retrieved from diverse environments such as freshwater and engineered systems, including anammox and AD reactors, encode a similar potential for algae-derived polysaccharides as average marine *Planctomycetota*, suggesting they are equally well-suited to targeting algal biomass. Across the phylum, we also detected GH families 29, 107, 139, 151 and 168, as well as other polyspecific families such as GH95, and GH141 that may target diverse sulphated fucan-based polysaccharides e.g. fucoidans, primarily found in various species of brown seaweeds [52] as well as other fucose-containing oligosaccharides (Fig. 3a). Although some members of the *Planctomycetota* phylum are already well-known utilisers of sulphated compounds including carrageenans and fucoidans [20, 53], little is known about the planctomycetotal enzymatic systems involved in the degradation of algal biomass in general. For instance, the complexity of fucoidans pressure bacteria to possess highly specialised enzymatic systems in order to fully degrade them, as described in *‘Lentimonas’* sp. CC4 [52]. Arguably, *Planctomycetota* might also be a key player in the degradation of various structurally complex fucoidans, given the widespread distribution of CAZymes targeting the backbone of sulphated polysaccharides in their genomes.

### Diversity of encoded GHs in individual *Planctomycetota*

We next examined the potential hydrolytic capacity of individual microorganisms by looking at the diversity profiles of CAZymes in genomes (number of distinct CAZyme families) assigned to the same phylogenetic class (Fig. 3b) and order (Fig. 3c; only GHs shown). The high diversity of CAZymes points to an extended capacity of the microorganisms to hydrolyse a wide range of complex polysaccharides in diverse environments [54]. Therefore, members of the unclassified UBA11346 class, planctomycetial (*Isosphaerales, Pirellulales*) and phycisphaeral (*Sedimentisphaerales, Tepidisphaerales*) orders demonstrate the largest potential to target diverse polysaccharides. The highest diversity of GHs is attributed to the UBA11346 putative class (38 ± 8 GHs per genome) while the lowest GH-encoding potential (between 4 and 13 different GHs) is typical for members of *Brocadiae* and the other yet unclassified classes (Fig. 3b; Additional File 2, Table S5). Comparing members at the order level, genomes assigned to *Sedimentisphaerales* (*Phycisphaerae* class) encode the highest number of hydrolysing enzymes assigned to GH and PL families, with an average of 47 ± 19 and 7 ± 4 distinct subfamilies per genome, respectively (Fig. 3c; Additional File 2, Table S5). Specifically, genomes assigned to the SG8-4 putative family of *Sedimentisphaerales* are characterised with one of the highest GH and PL diversities of all the *Planctomycetota* (Additional File 7, Fig. S3). The *Sedimentisphaerales* order also encodes up to 11 distinct GH families putatively targeting a different backbone of lignocellulosic polymers acting as “endo”- or “exo” enzymes (Additional File 7, Fig. S4). In comparison, most of the planctomycetotal genomes are characterised with at maximum six distinct CAZymes targeting the backbone of lignocellulose. Commonly found in terrestrial habitats *Isosphaerales* (*Planctomycetia* class; GH diversity 32 ± 16) and *Tepidisphaerales* (*Phycisphaerae* class; GH diversity 41 ± 11) also show multiple distinct GH modules, indicating their capacity to degrade diverse carbohydrates (Fig. 3c). The genomes assigned to the *Pirellulales* and *Gemmatales* orders correspond to the diversity of subfamilies in the range of 27 ± 15 and 15 ± 6, respectively. *Pirellulales* genomes assigned to the *Pirellulaceae* family are characterised with a much higher GH family diversity than the order average and could represent interesting “outliers” possibly targeting a wider range of polysaccharides (Additional File 7, Fig. S3). Furthermore, *Pirellulales* among all the *Planctomycetota* encode the highest number of GHs and PLs targeting algal carbohydrates, i.e. up to 13 different families (Additional File 7, Fig. S4).

### Diversity of accessory modules and rare CAZymes

**Fig. 4:**
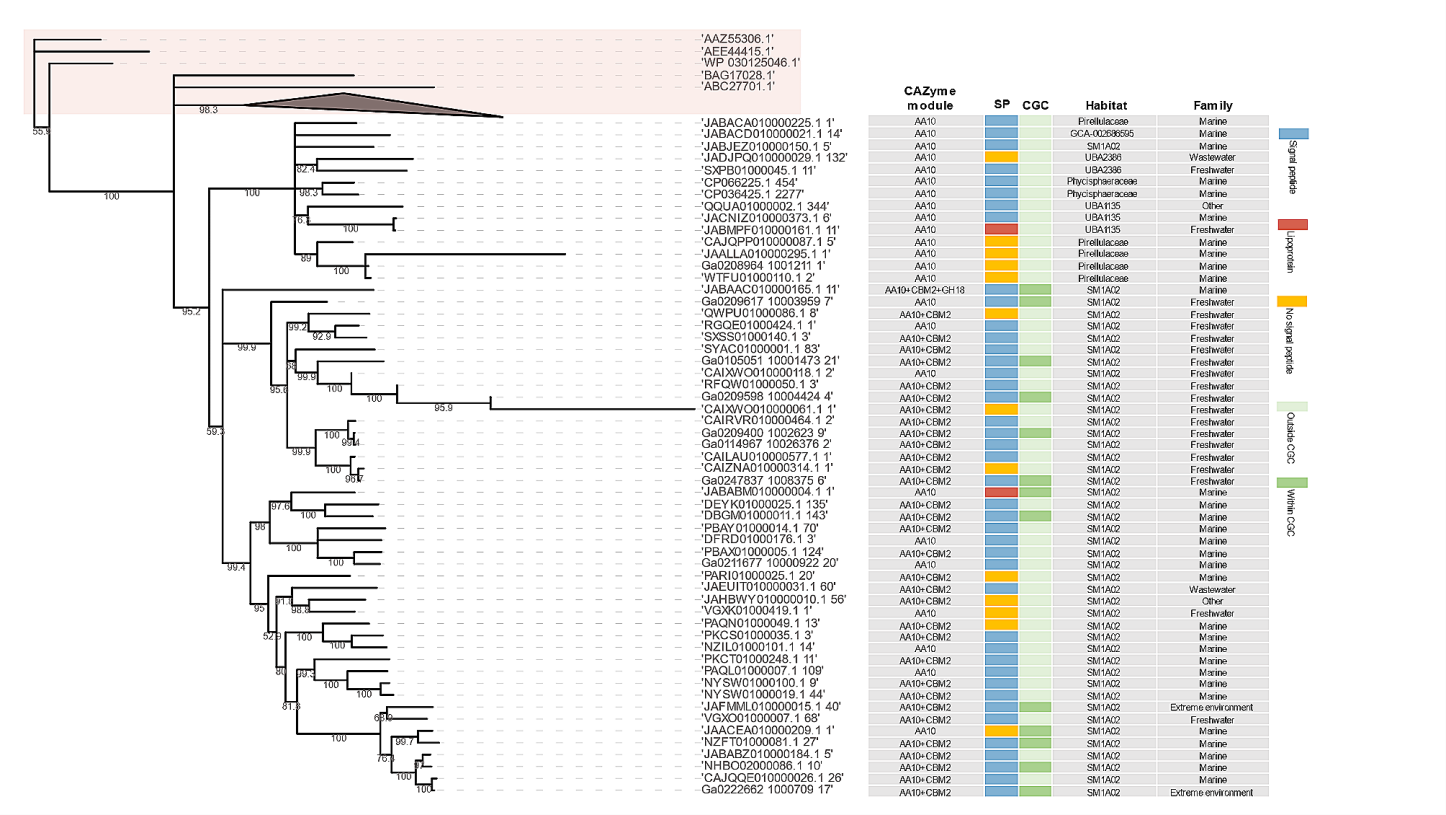
The phylogeny of planctomycetotal AA10 protein sequences retrieved from *Planctomycetota* genomes and the additional 15 AA10 protein sequences from bacteria representing other phyla with the LPMO activity described (highlighted in light red box). Bootstrap values are shown on branches. Additional metadata for planctomycetotal AA10 are presented: multi-modularity, presence of signal peptides, occurrence in CAZyme Gene Clusters (CGCs), taxonomic assignment and environmental origin (habitat)

Compared to other CAZyme classes, AAs are only occasionally detected in planctomycetotal genomes (Fig. 3b). Nevertheless, certain representatives of the *Planctomycetia* class encode up to six different AA families, including putative lignin peroxidases from the AA2 family (Additional File 7, Fig. S1). However, the AA2 family is mainly encoded in unclassified *Planctomycetota* from the UBA8742 and UBA1135 classes, equipping these members with a plausible capacity to degrade lignin and lignin derivates. All LPMOs identified are assigned to the AA10 family, which is the only LPMO family present in bacteria. They are mainly detected in genomes of uncultured members of the phycisphaeral SM1A02 putative family, and sporadically in other members of the phylum (Fig. 4). Importantly, SM1A02 genomes are particularly enriched in rare CAZymes e.g., present in < 5% of all planctomycetotal genomes. Of the 223 SM1A02 genomes used in our study, we identified 132 distinct CAZyme families, which corresponds to a wide diversity of CAZymes just within one family of bacteria, while per genome, on average only six different CAZyme families are encoded (Additional File 7, Figure S3). The representatives of the *Planctomycetia* class display a higher diversity of CE families than other *Planctomycetota* (Fig. 3b), encoding on average from 7 to 9 different CE families per genome. Overall, *Planctomycetales* and *Pirellulales* trend towards a higher number of esterases, including CEs and sulphatases (Additional File 7, Fig. S5), which are critical enzymes for debranching algal polysaccharides. Considering a rich selection of algae-degrading enzymes of *Pirellulales*, as well as a high number of CEs and sulphatases encoded in their genomes, we could infer the presence of a system designed to scavenge the algal biomass, reinforcing the earlier observations [55].

### Multi-modularity of planctomycetotal CAZymes

The variety of CBM modules seems to well reflect the diversity of GH families in some planctomycetotal genomes (Additional File 2, Table S6). Accordingly, planctomycetial *Isosphaerales* (rho = 0.70) and phycisphaeral *Sedimentisphaerales* (rho = 0.82), UBA1845 (rho = 0.76) and SM23-33 (rho = 0.70) show a strong correlation (*p* < 0.05) between GH and CBM family diversity (number of encoded GH and CBM families). However, members of *Tepidisphaerales*, characterised with one of the highest GH family diversities, do not follow this trend (rho = 0.21, *p* = 0.49). High correlation values could also result from some GH enzymes containing additional domains that accommodate other CAZyme modules, including CBMs, forming multi-modular enzymes [6]. For instance, members of *Sedimentisphaerales* encode on average 10% of CAZymes with multi-modular characteristics, including the highest number of unique module combinations (Additional File 7, Fig. S6). The most common combinations for all *Planctomycetota* are two-module CAZymes, but certain genomes encode regularly CAZymes with three and more modules (Additional File 2, Table S7). Although GHs are commonly associated with CBMs in different bacteria [6], the occurrence of complex CAZymes containing other enzymatic modules is relatively less frequent. However, such complexity may represent an adaptation strategy in competitive environments [56]. Of particular interests are CAZymes with four or five modules, encompassing a variety of endo-acting polysaccharides, including cellulases GH5 and GH9, as well as xylanases GH10 and GH62. These CAZymes often feature diverse appended CBMs, sometimes occurring in multiple instances, and are encoded by *Gemmatales, Pirellulales* (mainly cellulases) and *Sedimentisphaerales* (mainly xylanases).

Multiple catalytic domains within single polypeptides suggest that individual enzymes might independently target and degrade different components of the biomass, likely improving its overall hydrolysis rate, thus representing biotechnologically relevant targets [57, 58]. Previously, various bacteria have been demonstrated to secrete multi-modular CAZymes, either as free or membrane-bound enzymes, capable of acting on a diverse array of complex substrates [59, 60]. This includes modular cellulases featuring multiple catalytic domains (such as GH5 and GH9) along with non-catalytic domains, representing a novel arrangement distinct from the cellulosome expressed by some well-known cellulolytic microorganisms [61]. Unfortunately, the only planctomycetotal CAZyme characterised so far is a unimodular cellulase belonging to GH44 encoded by *Telmatocola sphagniphila* SP2T (*Gemmatales*) [62]. Therefore, the need for characterised multi-modular enzymes persists, and members of *Planctomycetota* hold promise for future discoveries.

### CAZyme gene coding frequency varies based on phylogeny and genome size

The *Planctomycetia* class has the largest genomes of all *Planctomycetota* (5.8 Mb on average; Fig. 1d) while phycisphaeral and brocadial genomes are among the smallest (3.6–3.9 Mb on average). However, regardless of the different genome sizes, members of the *Phycisphaerae* and *Planctomycetia* classes display similar CAZyme coding frequencies (Fig. 3b). A higher number of functionalities encoded in bacterial genomes was proposed to make up the larger genome size, shifting the potential for the discovery of new functionalities towards bigger genomes [63]. Previously, a positive correlation between the planctomycetotal genome size and the number of biosynthetic gene clusters (BGCs) was observed [22] that could not be extrapolated to their carbohydrate degrading potential. Here, we observed an unexpected tendency of *Planctomycetota* to discriminate between two main trends (T1 and T2), owing to the different number of encoded GHs and CBMs among the bacteria within the same range of genome size (Fig. 3d). The number of AAs, CEs and PLs simply correlates with the genome sizes as expected (*R* = 0.73, *p* < 0.01) and does not follow the aforementioned trends. Planctomycetotal genomes characterised with T1 trend, rich in CAZymes, represent a subset of microorganisms from AD, animal digestive tract and extreme environments which were previously recognised as promising sources for the discovery of biomass-degrading enzymes [9, 64, 65]. Considering individual genomes, the highest CAZyme coding frequency was attributed to an uncultivated member of the *Thermoguttaceae* family within the *Pirellulales* order (9.1%), followed by the SG8-4 putative family genome (8.7%) and an *Anaerohalophaeraceae* member (8.3%), with the latter two representing *Sedimentisphaerales* (Additional File 2, Table S8). These genomes were retrieved from the ruminant gastrointestinal tract system (*Thermoguttaceae*) and lab-scale anaerobic digestion studies (*Sedimentisphaerales*), respectively. Overall, there is a tendency for microorganisms inhabiting animal digestive systems to encode a significant fraction of CAZymes in their genomes, likely reflecting the adaptation and response to the diversity of dietary polysaccharides present in these environments [66]. In AD environments, the similarity in patterns is likely due to the presence of complex carbohydrates within their organic matter.

### The potential for the discovery of novel and unique CAZymes in *Planctomycetota*

At present, planctomycetotal CAZymes remain largely uncharacterised and to further prospect new functionalities in planctomycetotal genomes we evaluated the novelty of CAZymes by comparing their sequences to the entries in the CAZy database [5]. Overall, CAZymes encoded in *Planctomycetota* are distantly related to other bacterial CAZymes with the protein sequence identity ranging on average between 40% and 60% (Fig. 3e). A relatively large number of planctomycetotal CAZymes show very low sequence identity to any previously characterised enzyme, i.e. below 30% (Additional File 2, Table S9). For example, among the CBM modules with the lowest sequence identity are versatile CBM51 and CBM57, rhamnose-binding GH67, fucose-binding CBM47 and cellulose-binding CBM9 and CBM16. Below the set threshold we found only a single PL8 family, putatively involved in the breakdown of various polysaccharides such as xanthan, chondroitin sulphate, alginate, and CE15 which typically displays ligninolytic activity by cleaving ester bonds between lignin and hemicellulose components (CAZy database). The planctomycetotal AA10 are also distantly related to other currently described LPMOs and accordingly, their sequence similarity to other publicly accessible proteins is assessed at between 28% and 68% (Additional File 7, Fig. S7). Furthermore, the protein sequence alignment of all the planctomycetotal AA10 proteins revealed only moderate coverage in a few regions (pairwise identity median of 25.8%), advocating for high intra-specialisation within this group (Additional File 2, Table S10).

The high degree of novelty within planctomycetotal CAZyomes is in line with a previous study analysing a large group of β-galactosidase homologues from planctomycetotal genomes, which highlighted the presence of multiple, poorly characterised CAZymes, almost exclusively present in the PVC superphylum and some *Bacteroidota* [67]. Another recent study described the diversity of α-l-arabinofuranosidase homologues (GH51) from subantarctic intertidal sediments in different bacteria including *Planctomycetota* [68]. Similarly, further investigation of unclassified CAZymes, and CAZymes with a low sequence homology to known proteins, shall, in the future, allow the discovery of novel CAZyme functionalities as highlighted in the past by Naumoff and Dedysh [69].

Finally, we also evaluated the abundance of what we called unclassified GHs, PLs, and CEs, that is, enzymes which were not classified to any of the currently recognised CAZyme families (see Methods). We revealed that *Planctomycetota* typically encode between 1 and 5% of unclassified CAZymes in their genomes (Fig. 3f). Currently, comprehensive research data covering all bacterial phyla are not available, which prevents us from placing these findings within the broader context.

### Potential strategies for complex polysaccharide deconstruction – clustering of CAZymes in planctomycetotal genomes

**Fig. 5:**
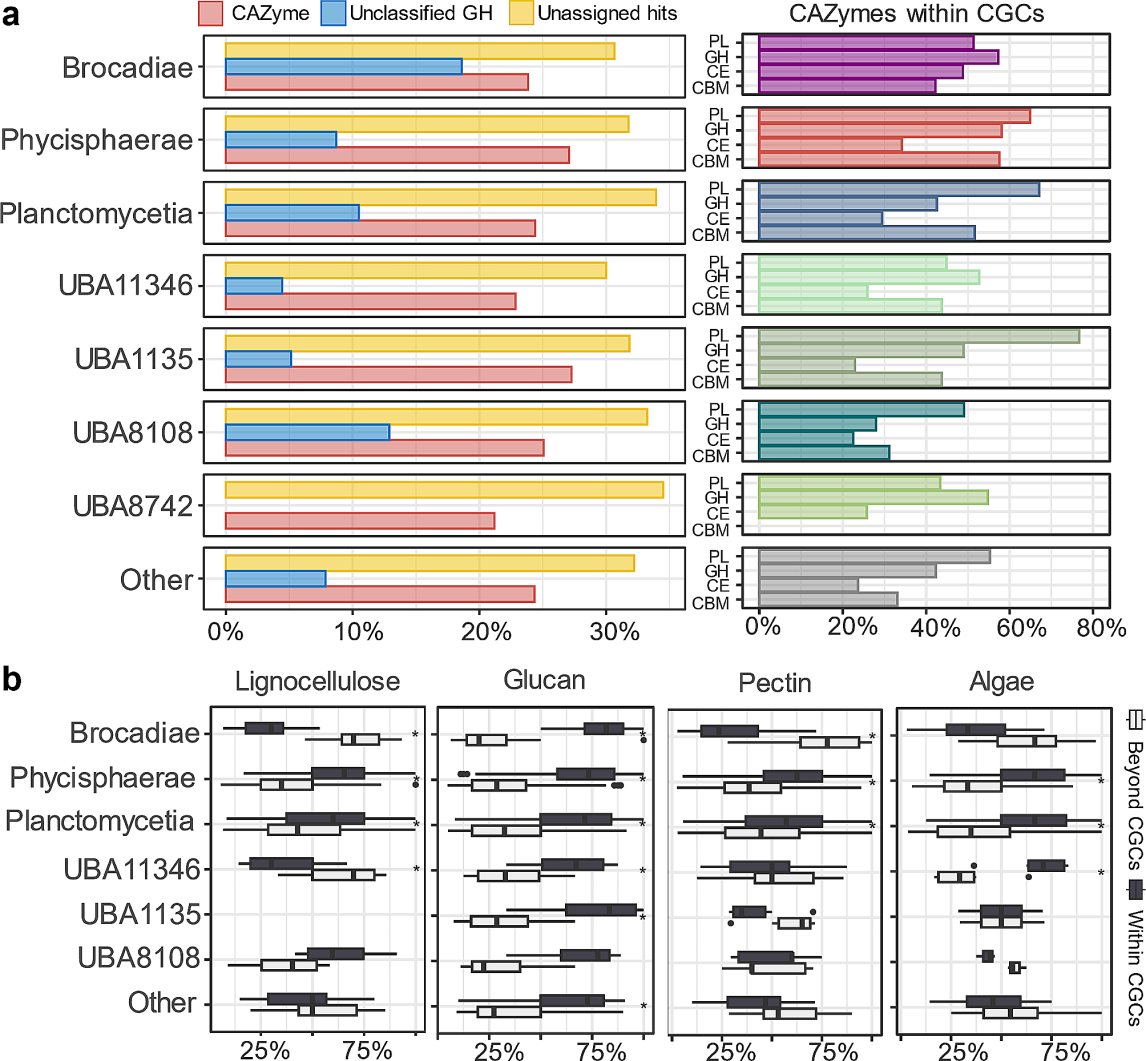
The CAZymes found within planctomycetotal CGCs. (**a**) Left panel: A mean fraction of hypothetical proteins, unclassified GHs and all CAZymes within CGCs for each planctomycetotal class. Right panel: A mean fraction of CAZyme coding genes co-localised within CGCs for each planctomycetotal class. (**b**) Ratio of functionally assigned CAZymes within or beyond CGCs in the individual genomes, estimated for the *Planctomycetota* classes. In light grey, CAZymes outside CGCs

Certain bacteria tend to cluster their CAZymes with complementary functions into so-called CAZyme gene clusters (CGCs) [66]. The most widely studied example is the polysaccharide utilisation locus (PUL) of *Bacteroidota* [70–72], however, similar gene clusters were also discovered in other bacterial phyla [73]. To the best of our knowledge, CAZyme clusters have not yet been characterised functionally in *Planctomycetota* although distinct groups within this phylum frequently encode co-localised CAZymes (Fig. 5). Most members of the *Brocadiae*, *Phycisphaerae* and putative UBA11346 classes co-localise more than 50% of their GHs on average. For comparison, up to 51% of predicted GHs are clustered in *Bacteroidetes cellulolysiticus*, which represents one of the highest scores in the bacterial domain [50]. Knowing that some planctomycetotal genomes in our database are incomplete and fragmented (Fig. 1b), the predicted number of CAZymes falling within gene clusters is likely a conservative estimate. We also looked at the portion of hypothetical genes as well as unclassified CAZymes within CGCs that could potentially represent novel enzymatic functions. Planctomycetotal orders encode between 30% and 34% of unassigned hits within CGCs that were not classified either as CAZymes or as regulatory/transport proteins (Fig. 5a). In general, research focusing on the functional characterisation of PULs, including analysis of genes previously categorised as hypothetical, can reveal new biocatalysts or led to the establishment of completely novel CAZyme families [74–76]. Further investigation of comparable systems in *Planctomycetota* is of high priority.

The CAZymes targeting different fractions of lignocellulose are regularly found within CGCs in almost all planctomycetotal classes, except for some unclassified UBA11346 and “other” class members, whose genomes show significant co-localisation only for glucan-targeting CAZymes, likely involved in the cellular metabolism (Fig. 5b). Among unclassified *Planctomycetota*, members assigned to UBA11346 deserve some attention. Despite the limited number of genomes in our database (only 16), they show a wide diversity and high coding frequency of CAZyme families, and their CAZyme distribution beyond CGCs suggest potentially a different enzymatic strategy to other generalist *Planctomycetota*. While genomes of *Isosphaerales* and *Pirellulales* have frequently co-localised diverse CAZymes, *Gemmatales* and *Planctomycetales* do not encode significantly more co-localised lignocellulolytic, pectinolytic and algalytic CAZymes (*p* < 0.01) than other orders (Additional File 2, Table S11). Finally, we also examined the putative CAZyme clusters involving LPMO coding genes and found that in most genomes, i.e. 80%, AA10 is not found within CGCs (Fig. 4).

### Cellular localisation of planctomycetotal CAZymes

**Fig. 6:**
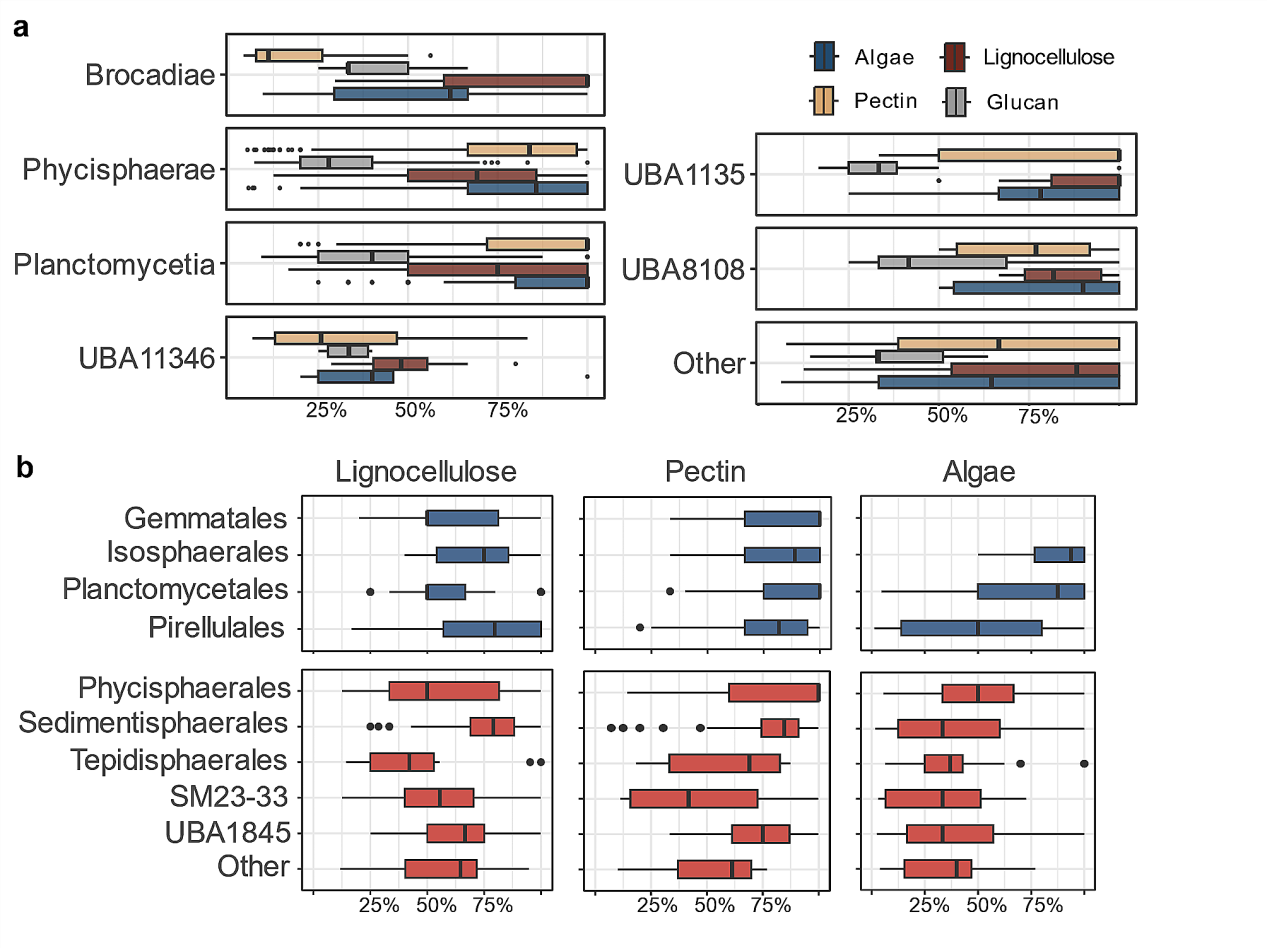
Predicted localisation of CAZymes putatively engaged in the degradation of specific polysaccharides, in the individual planctomycetotal genomes. (**a**) Ratio of CAZymes with any type of predicted signal peptide illustrated for the *Planctomycetota* classes. (**b**) Ratio of CAZymes with any type of predicted signal peptide illustrated for the *Planctomycetia* (blue) and *Phycisphaerae* (red) orders

To estimate the potential secretion of planctomycetotal CAZymes, either extracellularly or membrane-bound, we verified the presence of signal peptides in their enzymes [77]. The majority of *Planctomycetota* representatives were predicted to harbour N-terminal SPI (Sec) or Twin Arginine Transport (TAT) pathway signal peptides in more than 50% of their CAZymes (Additional File 7, Fig. S8). Phylum-wise, almost all of lignocellulose-, pectin- or algae-targeting enzymes are putatively secreted while CAZymes targeting α-glucans are much less common to incorporate signal peptides, mainly expected to be geared towards internal metabolism (Fig. 6a). Representatives of *Sedimentisphaerales, Pirellulales*, and *Isosphaerales* encode on average 76.1% ± 16.9, 74.4% ± 21.9, 72.9% ± 18.4 of their lignocellulolytic CAZymes as putatively extracellular enzymes, respectively (Fig. 6b). Differences between taxonomic classes could also be observed in pectinolytic and algalytic potentials. Members of *Planctomycetia*, including the *Planctomycetales* (85.7% ± 19.9), *Gemmatales* (82.2% ± 22.8) and *Isosphaerales* (81.6% ± 20.4) orders, encode the highest ratio of putatively secreted pectinases. Despite a low number of CAZymes targeting algae in the genomes belonging to *Isosphaerales*, they are predicted to be localised extracellularly (85.5% ± 18.8), while *Pirellulales* would putatively secrete half of the encoded algalytic repertoire (49.7% ± 33.8), on average.

Extracellular enzymes play an important role in initiating the hydrolysis of complex carbohydrates to shorter oligosaccharides, ready for cellular uptake [78]. Enzyme-secreting bacteria are beneficial to the whole community, as they pre-degrade larger fibres into smaller components which can be used by other microbes [79, 80]. Although in our analysis most of the planctomycetotal genomes flank their CAZymes with signal peptides to indicate the export of the proteins for the extracellular degradation, it was suggested that certain *Planctomycetota* selfishly import marine polysaccharides via an unknown mechanism [53]. In such cases, CAZymes flanked with the N-terminal peptide would only be transported to the periplasm, where the main saccharification would take place. Looking at the other types of signal peptides, we predicted that most of the planctomycetotal classes also encode in their genomes CAZymes with lipoprotein signal peptides cleaved by Lsp (leader peptidase or signal peptidase II; Additional File 7, Fig. S8). This type of signal peptide often serves for intracellular localisation [41], thus putatively supports the anchoring of their enzymes to either the inner or the outer cell membrane. The membrane anchored extracellular CAZymes would benefit the host more than the other community members, allowing the higher share of the liberated oligosaccharides to be taken by the main enzyme producer. An interesting feature, exclusive to *Planctomycetota*, was further observed by Boedecker et al. [12], who described an extreme enlargement of the periplasmic space in *Planctopirus limnophila* (*Planctomycetales* order), accompanied by its ability to bind sugar moieties using crateriform structures when feeding on complex, branched glucan (dextran). Likewise, type IV pili of *Fimbriiglobus ruber* (*Gemmatales* order) were shown to enhance bacterial adhesion to chitin and other biopolymers [81]. So far, these mechanisms have only been proven experimentally for some species from the *Planctomycetia* class, and any further ecological relevance, directly or indirectly related to polysaccharide uptake and degradation, remains to be scrutinised.

### Perspectives on biotechnological applications

There is a viable interest in exploring how microorganisms utilise polysaccharides, as understanding these mechanisms can help us not only to unveil their environmental interactions, but also reinforce the current solutions or develop new industrial technologies [56, 73, 82]. For instance, the arrangement of the CAZymes in clusters allows the coordination of gene expression, resulting in the protein ensembles required for a complex carbohydrate saccharification [70, 83, 84]. As such, the synergistically acting enzymatic complexes could be extracted together, simplifying the enzymatic cocktail design [85]. Similarly, the role of extracellular enzymes extends to various biotechnology applications, mainly as nature-inspired enzymatic cocktails that simplify the extraction and downstream processing [86, 87]. In view of this, we think that the as yet uncultured members of phycisphaeral *Sedimentisphaerales* and planctomycetial *Pirellulales* characterised with putatively extracellular, diverse and frequently co-localised CAZymes are among the high-priority targets for extending the strategies to be applied to biomass-based biorefineries. Nevertheless, the carbohydrolytic potential encoded by *Planctomycetota* cannot fully reflect their microbial capabilities in the environment and a deeper understanding of planctomycetotal metabolism and ecology is essential for their effective biotechnological application in the developing biorefinery sector. Contextualising the genomic measurements to metabolic traits remains difficult; yet, the novel approaches present a promising avenue for overcoming this challenge, enabling the accurate prediction of microbial phenotypes from genomic data exclusively [88]. It is worth noting that slow growth rates are a hallmark of *Planctomycetota*, exhibited by many isolated strains of this phylum [89], and one of the key considerations for developing specific applications lies in the continued optimisation of their growth. Culture-based methods are still required for optimising and scaling up biotechnological processes [90], posing an ongoing challenge, however a few methods have already been established for *Planctomycetota* [91, 92]. On the other hand, for the discovery of novel CAZymes, genomic approaches provide an alternative to culturing microorganisms [85].

Lignocellulose, despite being generally common and abundant resource, is an untapped biomass feedstock due to its recalcitrance [93]. In consequence, this so-called second-generation feedstock still lacks economic viability at the industrial scale and new approaches are needed to improve the enzymatic hydrolysis of diverse plant biomasses [87]. It has been suggested that LPMOs help degrade the recalcitrant lignocellulose fractions efficiently by boosting the overall activity of common GHs [94, 95]. Here, we identified LPMO coding genes in *Planctomycetota* genomes exhibiting high diversity of homology, which likely reflects their different origins or evolutionary history. Based on protein phylogeny, AA10 sequences retrieved from *Planctomycetota* form separate cluster than other, even phylogenetically unrelated bacteria representing different phyla such as *Actinomycetota, Pseudomonadota* and *Bacillota* (Fig. 4; Additional File 7, Fig. S7). Thus, as yet uncharacterised planctomycetotal AA10 family might represent new hydrolytic functionalities that are not only distantly related to existing sequences in public databases, but also functionally diverse. We argue that LPMOs co-localised with other GHs might represent an evolutionary optimised version of an efficient enzymatic machinery, possibly targeting complex polysaccharides like crystalline cellulose or chitin. However, while LPMOs might specifically enhance the activity of co-localised GHs, LPMOs encoded beyond clusters might be universal boosters helping diverse enzymes to attack glycosidic bonds within the polysaccharide moieties. Cellular investment in a single enzyme production would offer an interesting cost-saving strategy compared to the expression of the whole enzyme cluster. Thus, such potent LPMOs would represent an interesting component of industrially relevant enzymatic preparations that could significantly reduce the cost of the enzymatic biomass processing technologies, e.g. for bioethanol production [96]. So far, none of the planctomycetotal LPMOs have ever been analysed, therefore their enzymatic activities should be further studied to determine their effectiveness for biomass processing.

Algae, considered a third-generation biomass, could offer potential advantages over lignocellulose such as negligible presence of lignin, making it less resistant to degradation and reducing the need for intensive pre-treatments [97, 98]. However, the diversity of unique algal polysaccharides, particularly the recalcitrant fucoidans produced by brown algae, seem to become the major obstacle for developing biorefineries [52, 99]. It is therefore crucial to design an individual approach for each type of biomass, and nature-inspired cocktails seem to be a promising alternative for the complete conversion of different biomasses to fermentable sugars [100–102]. Initially, we expected the *Planctomycetota* retrieved from marine environments to serve as a reservoir for diverse algalytic CAZymes, due to the abundance of algal biomass in seawater. Contrary to our expectations, genomes of *Planctomycetota* retrieved from engineered systems such as AD, encode a similar algae-targeting potential to marine-sourced members of the phylum. Furthermore, their potential specialisation towards specific algal fractions such as fucan-based compounds, is particularly intriguing given the possibilities for its harnessing to develop well-defined applications. Overall, the capacity of anaerobic microbes to degrade algal biomass directly in AD reactors opens up a new perspective for its valorisation in the context of biogas production and the development of biorefineries. As the field of green biotechnology continues to advance, the interest in the planctomycetotal-based applications is likely to grow.

## Conclusions

The *Planctomycetota* phylum offers a wealth of diverse, novel CAZymes of potential industrial interest. Our study provides a new perspective on the planctomycetotal carbohydrolytic potential, highlighting the presence of distinct phylogenetic groups with both general and specialised abilities to break down complex carbohydrates. We identified planctomycetotal families affiliated to the *Sedimentisphaerales* and *Pirellulales* orders that are not yet well characterised as suitable candidates for applications in second generation biomass transformation technologies, due to their diverse CAZymes, including extracellular lignocellulose targeting enzymes. In addition, we showed that some *Planctomycetota* possess LPMOs, which can be further employed to boost the overall activity of GHs in lignocellulose hydrolysis. To our surprise, AD-sourced *Planctomycetota* appeared to be well-equipped for degrading algal-derived polysaccharides, thus representing a perspective for a direct algal biomass transformation to bioenergy in methanogenic reactors. Overall, our findings have implications for directing bioprospecting ventures to enable a more effective discovery of CAZymes in *Planctomycetota*. Although the most interesting planctomycetotal models represent still uncultivated bacteria, their enzymes can already be explored for specific applications thanks to their identification and characterisation through *in silico* studies.

### Electronic supplementary material

Below is the link to the electronic supplementary material.

Supplementary Material 1

Supplementary Material 2

Supplementary Material 3

Supplementary Material 4

Supplementary Material 5

Supplementary Material 6

Supplementary Material 7

## Data Availability

All data generated or analysed during this study are included in this article and its supplementary information files. Accession numbers of public genomes used in this study are listed in the Additional File 1, Table S1. Remaining genomes from previous, own studies are available upon request.

## Abbreviations

AD: Anaerobic Digestion
CAZyme(s): Carbohydrate Active Enzyme(s)
CGC(s): Carbohydrate Gene Cluster(s)
GH: Glycoside Hydrolases
CE: Carbohydrate Esterases
PL: Polysaccharide Lyases
AA: Enzymes with auxiliary activities
PCoA: Principal Coordinates Analysis
