# Supplementary material for: Comparative genomic analysis of Planctomycetota potential for polysaccharide degradation identifies biotechnologically relevant microbes
Source: BMC Genomics. 2024 May 27;25:523. doi: 10.1186/s12864-024-10413-z (PMC11131199; doi:10.1186/s12864-024-10413-z)
Supplement: Supplementary file 7 — Supplementary Material 7 [file 12864_2024_10413_MOESM7_ESM.pdf]

Comparative genomic analysis of *Planctomycetota* potential for polysaccharide degradation identifies biotechnologically relevant microbes

Dominika Klimek[1,2], Malte Herold[1], Magdalena Calusinska[1]

1 Environmental Research and Innovation Department, Luxembourg Institute of Science and Technology (LIST), 41 rue du Brill, L-4422 Belvaux, Luxembourg

2 The Faculty of Science, Technology and Medicine (FSTM), University of Luxembourg, 2 Avenue de l'Université, L-4365 Esch-sur-Alzette, Luxembourg

Supplementary Figures

In this supplementary file, we provide figures that serve to elaborate on the discussion within the main text.

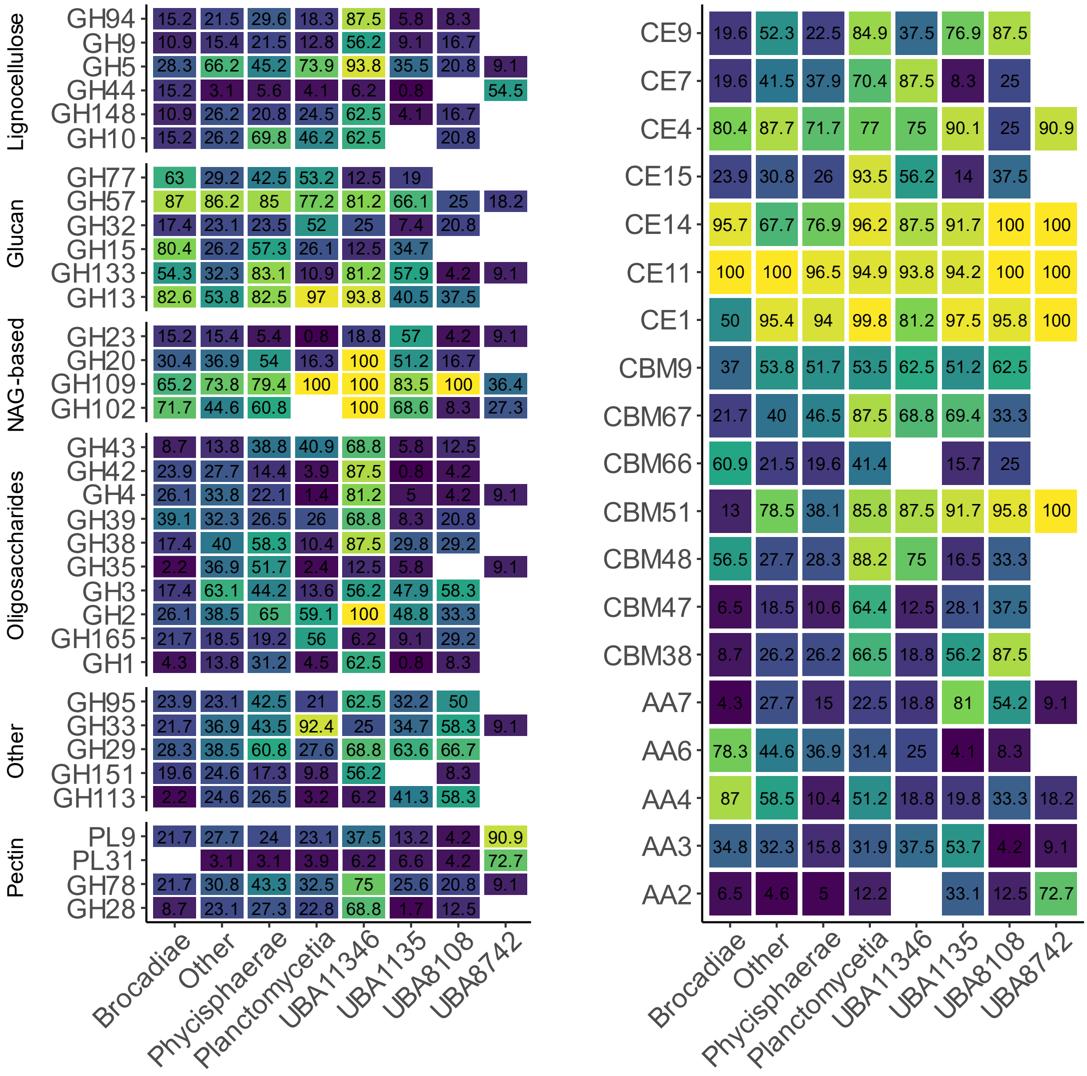

Fig. S1. The most commonly encoded CAZyme families within planctomycetotal classes (at least 50% of class representatives). Numbers represent a fraction of genomes encoding each CAZyme family (%) belonging to cleavage enzymes (GH and PL) and other class of enzymes (AA, CE, CBM).

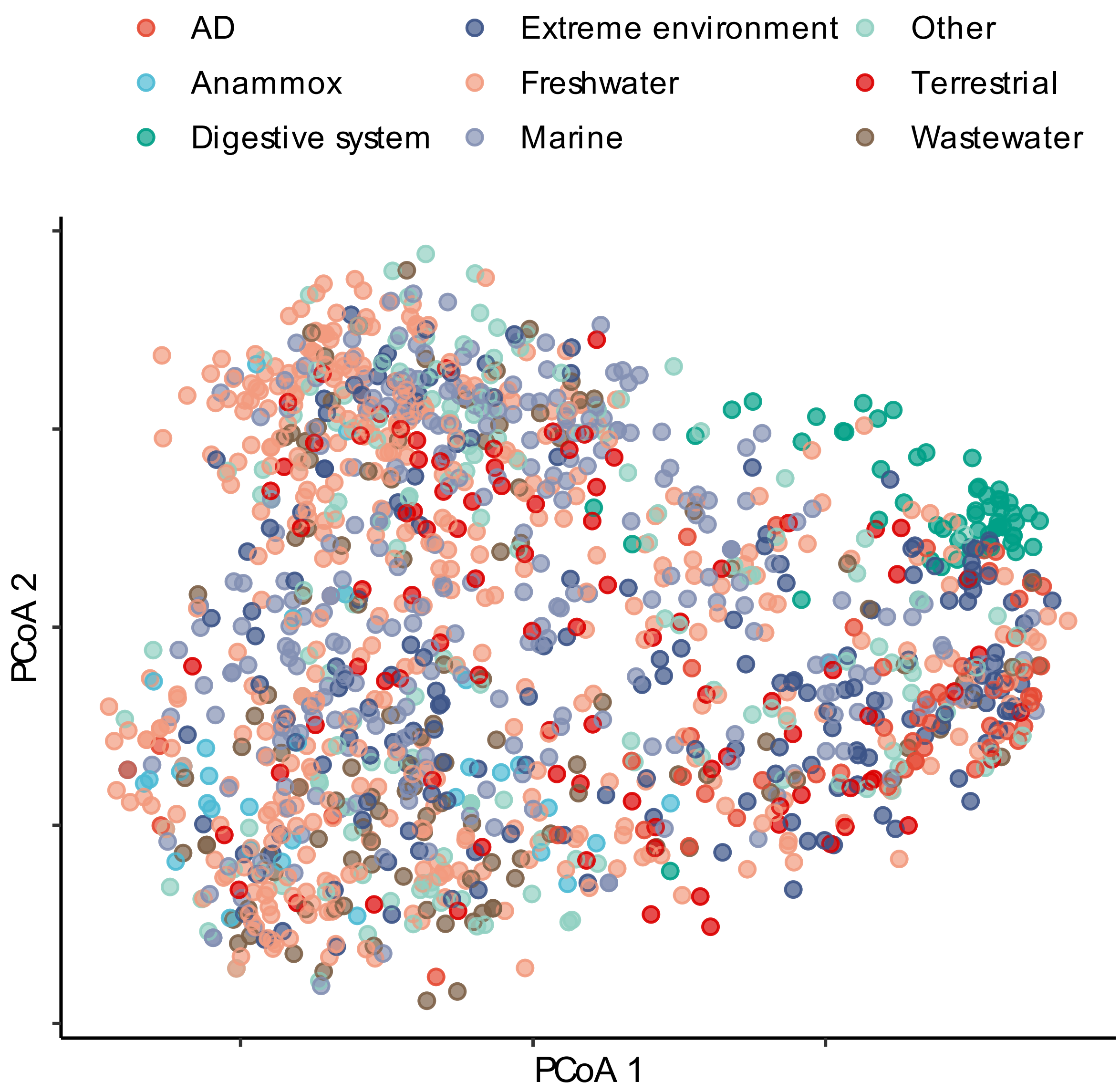

Fig. S2. Principal coordinates analysis (PCoA) ordination based on the Jaccard distance presence-absence matrix of GHs encoded in planctomycetotal genomes, coloured by habitat (environmental origin). We could observe a clear grouping of planctomycetotal genomes retrieved from animal digestive systems (coloured in green).

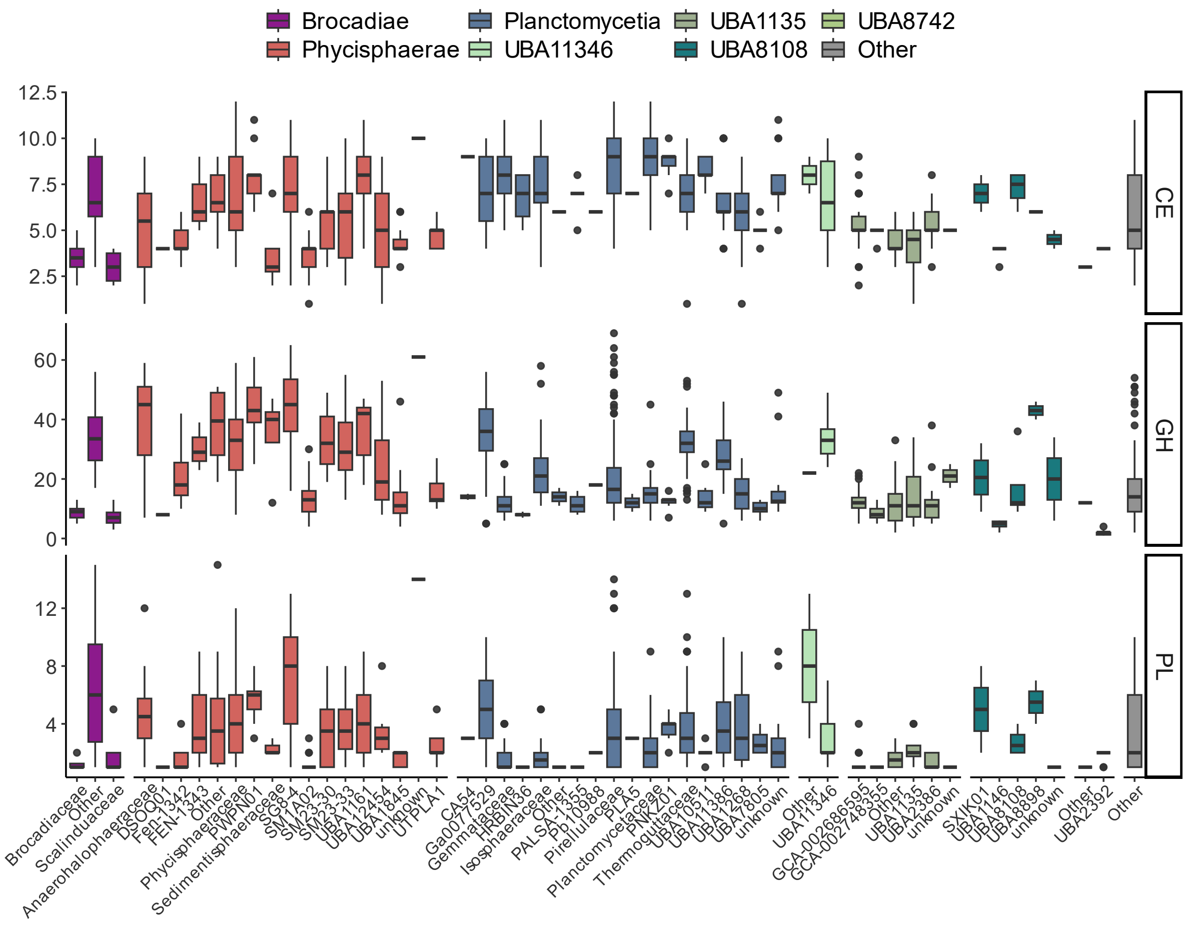

Fig. S3. CAZyme diversity of selected classes (CE, GH, PL), calculated for individual genomes of Planctomycetota, coloured by class affiliation and grouped at the family level.

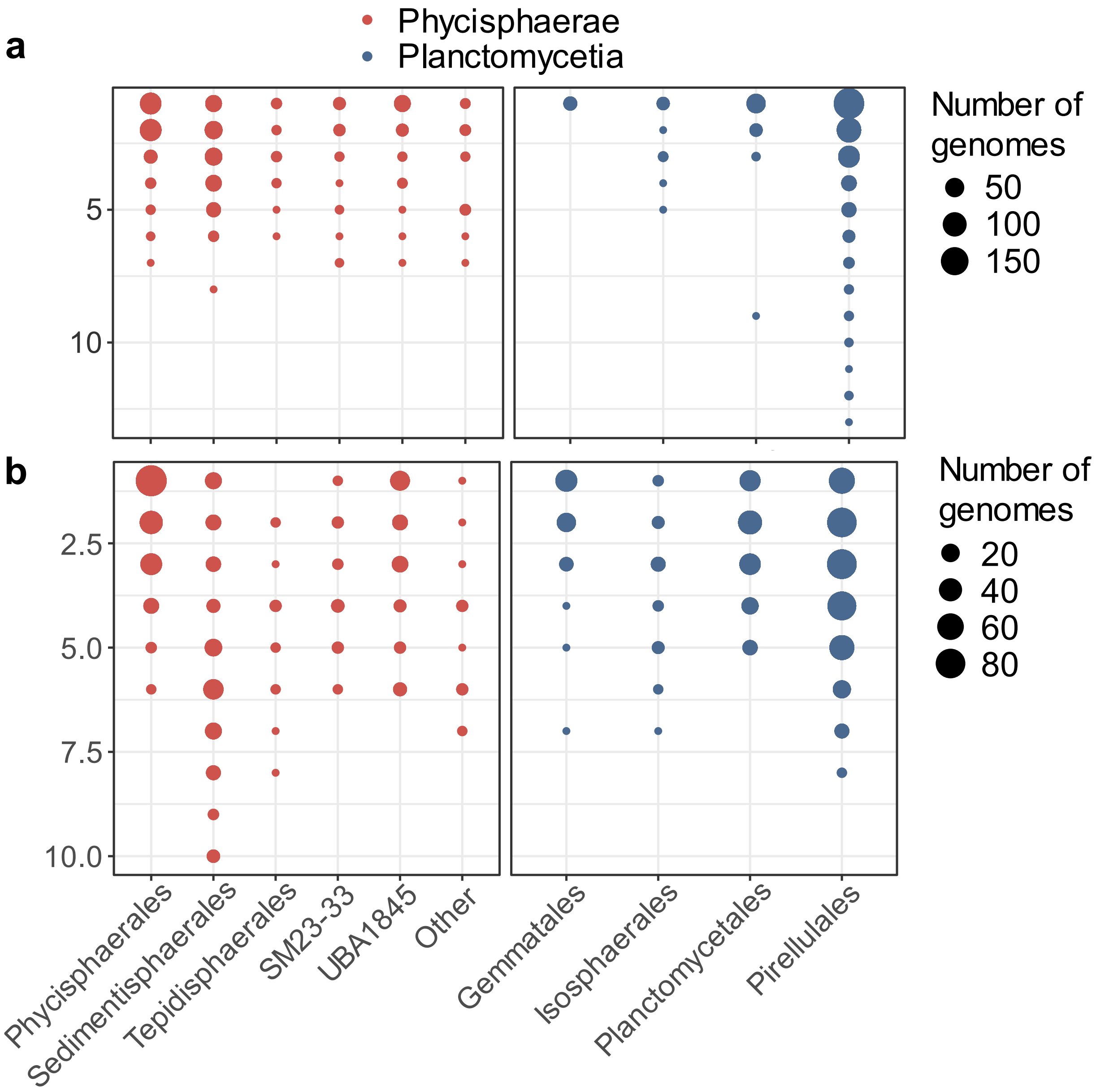

Fig. S4. The number of different GH and PL families putatively involved in (a) algae and (b) lignocellulose processing, calculated for order and coloured by class affiliation. Only *Planctomycetia* and *Phycisphaerae* classes are shown.

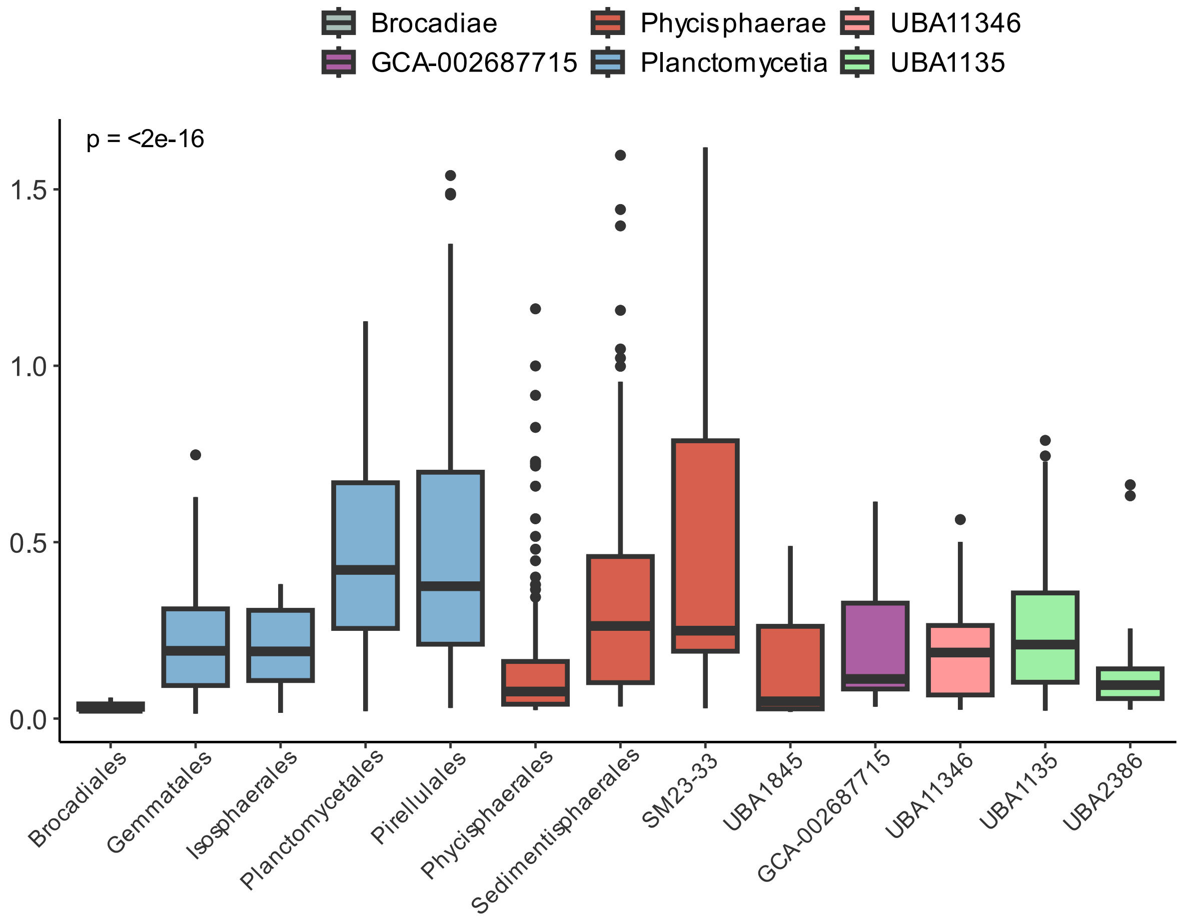

Fig. S5. Encoded percentage of sulfatases (EC number 3.1.6.-) in individual genomes, grouped at order level and coloured by class affiliation.

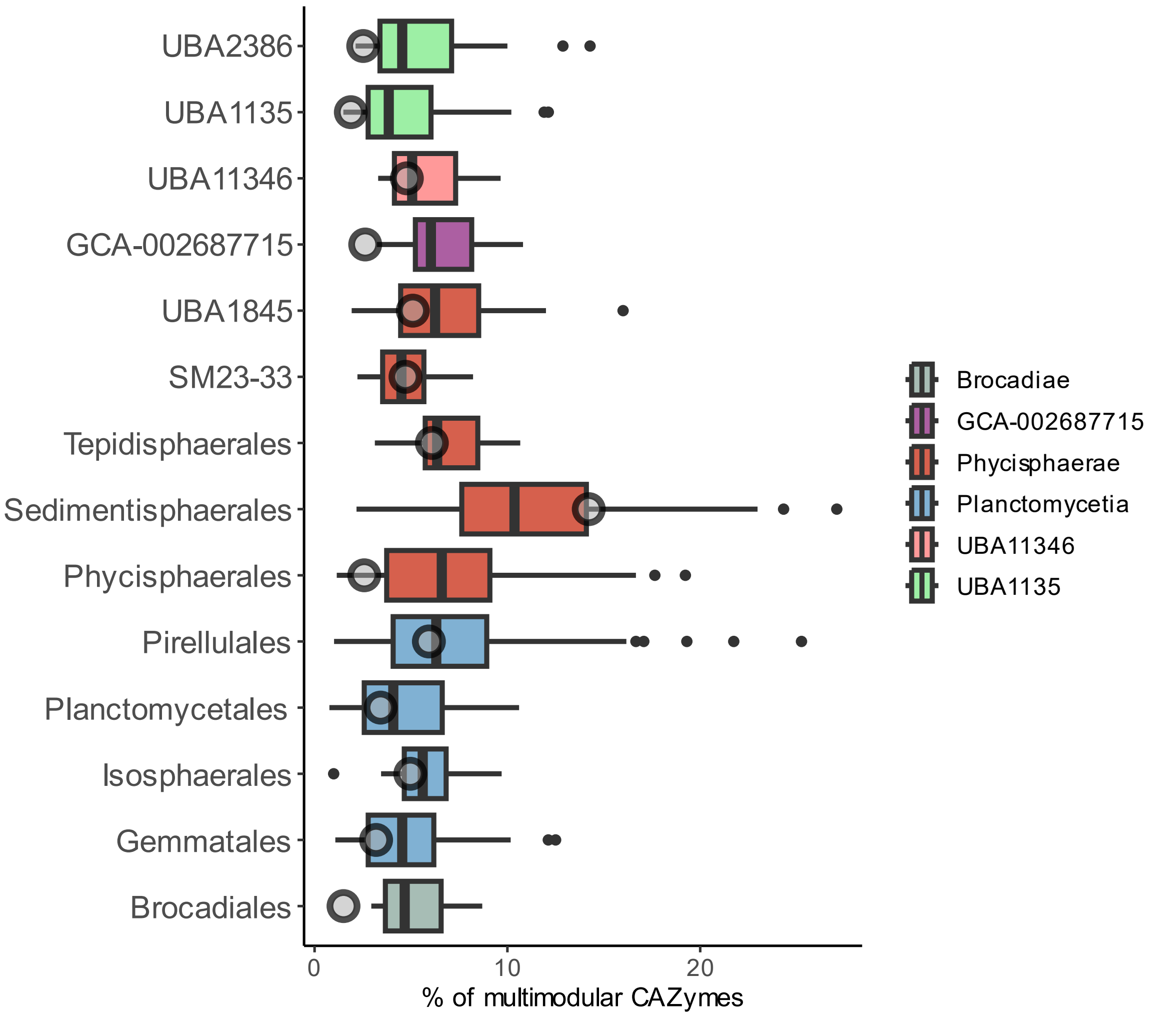

Fig. S6. The percentage of multi-modular CAZymes in planctomycetotal genomes. Multi-modular CAZymes consist of multiple modules with distinct functions, each contributing to the overall enzymatic activity. Boxplots show percentage of multi-modular CAZymes for each genome at order level coloured by class affiliation. Gray circles show the diversity of multi-modular CAZyme modules (number of unique CAZyme combinations).

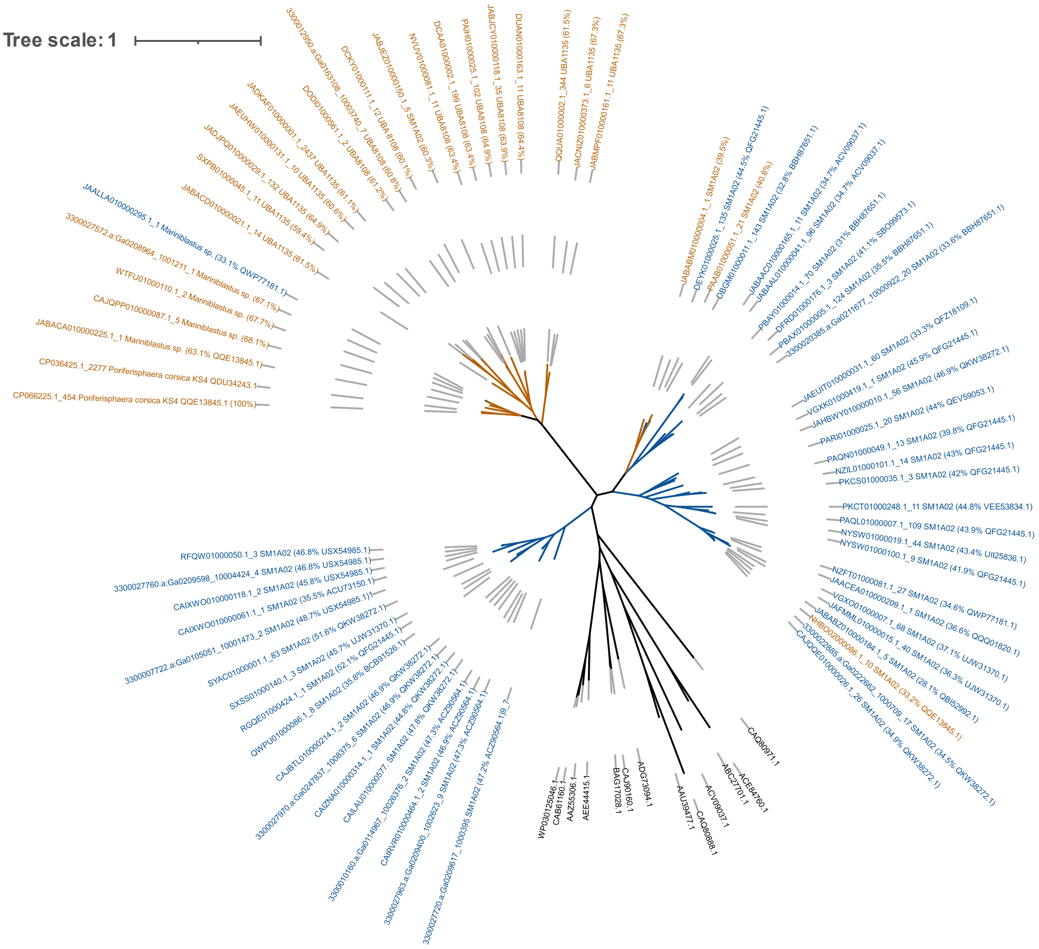

Fig. S7. Phylogeny of 57 planctomycetotal AA10 protein sequences (coloured in blue and brown) and 15 other AA10 enzymes with confirmed lytic oxidoreductase activity (coloured in black). Neighbor-Joining tree was built using aligned sequences by muscle algorithm. In brackets percentage identity of each protein and its closest blast hit (NCBI entry). Nodes coloured in brown represent proteins with the highest similarity to other *Planctomycetota* (*Poriferisphaera corsica* KS4*)*. Nodes coloured in blue point to other donor bacteria such as *Actinomycetota, Bacillidota* and *Proteobacteroidota.* Nodes coloured in black represent enzymes already described in the literature.

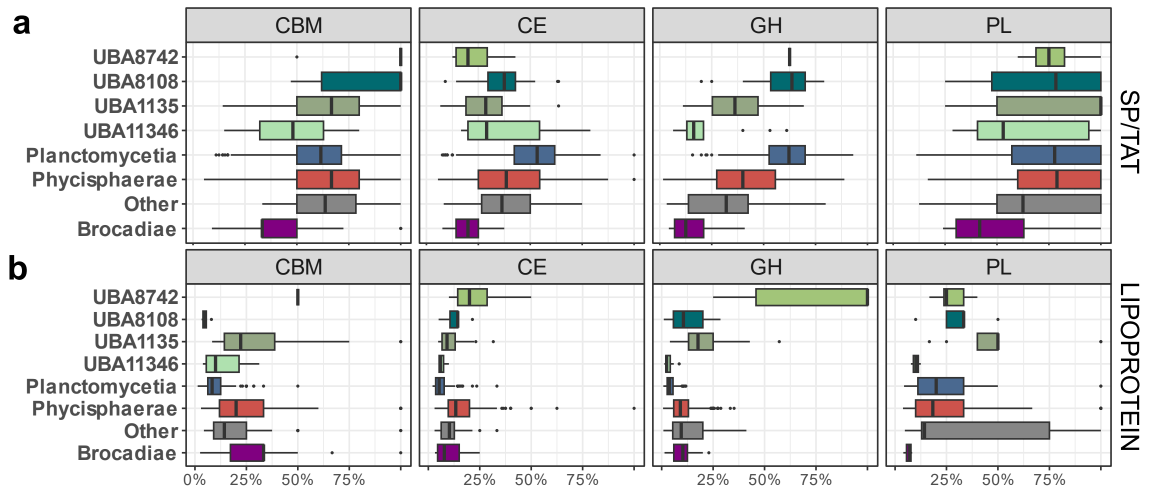

Fig. S8. Predicted localisation of CAZymes, coloured at the class level. Only main trends are shown (a) Ratio of CAZymes with predicted SP or TAT signal peptide for each planctomycetotal genome, grouped at the class level (b) Ratio of CAZymes with predicted signal peptides for lipoproteins for each planctomycetotal genome, grouped at the class level.
